# Engineering strategy of yeast metabolism for higher alcohol production

**DOI:** 10.1186/1475-2859-10-70

**Published:** 2011-09-08

**Authors:** Fumio Matsuda, Chikara Furusawa, Takashi Kondo, Jun Ishii, Hiroshi Shimizu, Akihiko Kondo

**Affiliations:** 1 Organization of Advanced Science and Technology, Kobe University, Rokkodaicho, Nada-ku, Kobe, Hyogo, Japan; 2 Department of Bioinformatic Engineering, Graduate School of Information Science and Technology, Osaka University, Yamadaoka, Suita, Osaka, Japan; 3 Department of Chemical Science and Engineering, Graduate School of Engineering, Kobe University, Rokkodaicho, Nada-ku, Kobe, Hyogo, Japan

## Abstract

**Background:**

While *Saccharomyces cerevisiae *is a promising host for cost-effective biorefinary processes due to its tolerance to various stresses during fermentation, the metabolically engineered *S. cerevisiae *strains exhibited rather limited production of higher alcohols than that of *Escherichia coli*. Since the structure of the central metabolism of *S. cerevisiae *is distinct from that of *E. coli*, there might be a problem in the structure of the central metabolism of *S. cerevisiae*. In this study, the potential production of higher alcohols by *S. cerevisiae *is compared to that of *E. coli *by employing metabolic simulation techniques. Based on the simulation results, novel metabolic engineering strategies for improving higher alcohol production by *S. cerevisiae *were investigated by *in silico *modifications of the metabolic models of *S. cerevisiae*.

**Results:**

The metabolic simulations confirmed that the high production of butanols and propanols by the metabolically engineered *E. coli *strains is derived from the flexible behavior of their central metabolism. Reducing this flexibility by gene deletion is an effective strategy to restrict the metabolic states for producing target alcohols. In contrast, the lower yield using *S. cerevisiae *originates from the structurally limited flexibility of its central metabolism in which gene deletions severely reduced cell growth.

**Conclusions:**

The metabolic simulation demonstrated that the poor productivity of *S. cerevisiae *was improved by the introduction of *E. coli *genes to compensate the structural difference. This suggested that gene supplementation is a promising strategy for the metabolic engineering of *S. cerevisiae *to produce higher alcohols which should be the next challenge for the synthetic bioengineering of *S. cerevisiae *for the efficient production of higher alcohols.

## Background

The bioproduction of higher alcohols with more than 3 carbon atoms is desirable because they have the preferred properties of renewable liquid fuels and wide applications in commodity chemicals. In designing fermentation processes for mass production, the development of recombinant microbial strains is a critical first step [1]. The pioneering achievements were demonstrated by the construction of metabolically engineered *Escherichia coli *strains that produce butanols and propanols. Due to insufficient genetic tools for the natural 1-butanol producer clostridia [2], *E. coli *has been engineered for 1-butanol fermentation by introducing the CoA-dependent clostridial pathway [3-7]. The production of 1-butanol was preliminary achieved in the range of 0.55 to 1.2 g/L [4,6,8]. The maximum 1-butanol titer (30 g/L) was achieved by the introduction of irreversible transenoyl-CoA reductase and the creation of NADH and acetyl-CoA driving forces [9,10]. The production of higher alcohols, including 1-propanol, 1-butanol, isobutanol, 2-methyl-1-butanol, and 3-methyl-1-butanol, was also demonstrated by introducing the keto-acid pathway [8,11-22]. This implementation resulted in 1-butanol production of approximately 0.85 g/L [11]. In these studies, the production of the target alcohols was achieved by the introduction of the foreign genes required for their biosyntheses and by gene deletions to modulate the entire metabolism of the strains.

**Figure 1:**
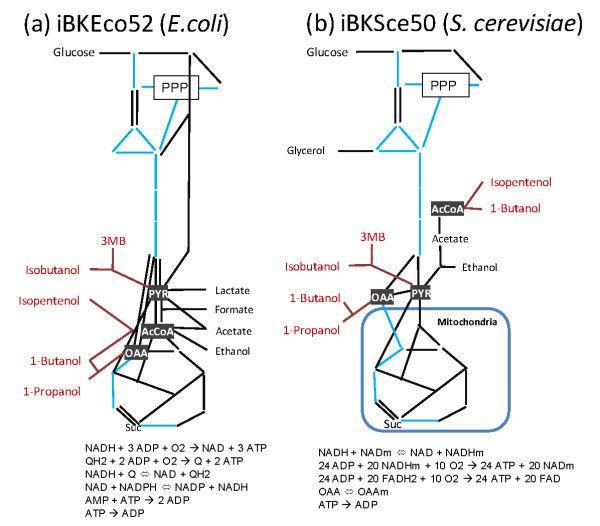
**Structures of central metabolic pathways in (a) *Escherichia coli *and (b) *Saccharomyces cerevisiae***. The backbone models for *E. coli *(iBKEco52: an iBioK*E. coli *metabolic model containing 52 reactions) and *S. cerevisiae *(iBKSce50) were reconstructed on the basis of these pathways. The black and blue lines represent irreversible and reversible reactions, respectively. The structure of the pentose phosphate pathway (PPP) is simplified in the figure. The metabolite for the carbon source (glucose) and fermentation products are described in plain black font. The metabolic pathways for the biosynthesis of each higher alcohol are shown in red. Abbreviations: AcCoA (acetyl-CoA), OAA (oxaloacetate), PYR (pyruvate), Suc (succinate), and 3 MB (3-methyl-1-butanol).

Based on these successes, a metabolic engineering study was initiated in baker's yeast (*Saccharomyces cerevisiae*). Yeast is a promising host for cost-effective biorefinary processes due to its tolerance to various stresses during fermentation. Tolerance to high concentrations of alcohol should be useful for the industrial production of higher alcohols [23-28]. The constructed yeast strains, however, exhibited limited production of higher alcohols. For instance, engineered *S. cerevisiae *expressing enzymes involved in the CoA-depending clostridial pathway yielded only 2.5 mg/L of 1-butanol [29]. One technical reason for the low yield is the reduced activity of the introduced enzymes due to the ectopic expression of the bacterial genes. Another possible reason is that there might be a problem in the structure of the central metabolism of *S. cerevisiae*. The structure of the central metabolism of *S. cerevisiae *is distinct from that of *E. coli *in terms of the anaplerotic pathways, cytosolic acetyl-CoA (AcCoA) biosynthesis, fermentation products, and occurrence of mitochondria (Figure 1). For the mass production of higher alcohols, these metabolic pathways must be modified to increase the metabolic flow towards the synthesis of the target alcohols. This means that the precursor synthesis, cofactor regeneration, and supply of the building blocks for active cell growth must be simultaneously achieved by the central metabolisms while maintaining the carbon and redox balances. Because the range of possible distributions of metabolic fluxes is restricted by the structure of the central metabolic network, *S. cerevisiae *may not be able to supply the required amount of the precursor and the reducing powers needed for the biosynthesis of higher alcohols. In such cases, the engineering strategy has to be revised by further modification of *S. cerevisiae *metabolism.

In this study, the potential production of higher alcohols by *S. cerevisiae *is compared to that of *E. coli *by employing *in silico *metabolic simulation techniques based on flux balance analysis (FBA). FBA is a mathematical approach for analyzing the flow of metabolites through a metabolic network [30-32]. The distribution of metabolic fluxes in the metabolic networks can be analyzed using the stoichiometric model of the metabolic reactions without knowledge of the metabolite concentrations or details of the enzyme kinetics [33,34]. Although FBA assumes the ideal metabolism conditions by ignoring all dynamic aspects of metabolic regulations such as gene expression, feedback regulation, and posttranslational modifications, it has been demonstrated that FBA can evaluate the performance of a metabolic network [30,33]. In this report, FBA-based metabolic simulation was demonstrated by following the strategy applied for the actual metabolic engineering. After reactions required for the biosynthesis of higher alcohols were introduced into the metabolic models, FBA-based metabolic simulations were performed for multiple gene-deletion mutants to evaluate the capabilities of the engineered strains to produce higher alcohols. For this purpose, the backbone metabolic models, which describe the essential structures of their central metabolism (Figure 1), were employed to reduce the number of gene-deletion combinations to be tested. By using the small backbone models, the performance of all single, double, triple, and quadruple deletion mutants could be comprehensively investigated within a realistic period. The *in silico *analysis was applied for the backbone models of both *E. coli *and *S. cerevisiae *to highlight differences between their metabolic networks. The comparative analyses revealed that the central metabolism of *S. cerevisiae *has limited potential for the efficient production of higher alcohols in comparison with that of *E. coli*. It is also predicted that its poor productivity can be improved by extension of the central metabolic network.

## Results

### Construction of backbone models

The backbone metabolic models of *E. coli *and *S. cerevisiae *were employed for this analysis. The backbone metabolic models, which describe the essential structures of the central metabolism, were constructed from information from both the literature [35-41] and databases [42-44] (Figure 1 and Additional file 1). The backbone models for *E. coli *and *S. cerevisiae *are hereafter designated as iBKEco52 (i.e., an iBioK*E. coli *metabolic model containing 52 reactions) and iBKSce50, respectively. In iBKSce50, the metabolic network is compartmentalized in the cytosol and mitochondria. Although the backbone models contain only 50 or 52 reactions, their behavior was similar to that of genome-scale models of *E. coli *(iJR904) and *S. cerevisiae *(iMM904)[45,46] (Additional file 2). These original models were modified by the addition of reactions for the synthesis of each higher alcohol. As shown in Figure 1, the following 6 pathways were tested: (1) 1-propanol by oxaloacetate (OAA), (2) 1-butanol by AcCoA, (3) 1-butanol by OAA, (4) isobutanol by pyruvate (PYR), (5) 3-methylbutanol by PYR, and (6) isopentenol from AcCoA (Additional file 3). By using these models, the potential production capabilities of higher alcohols were compared between *E. coli *and *S. cerevisiae*.

### Metabolic simulation of reaction-deleted mutants of *E. coli*

As the first step of the analysis, the metabolic performance of *E. coli *was evaluated as a reference for the comparison. The potential productions of higher alcohols by *E. coli *were determined by employing the FBA-based metabolic simulations. In the procedure, the metabolic flux distribution was optimized to maximize the cell growth rate [30]. It is based on the assumption that a microbial metabolic system should evolve towards faster cell growth [47]. As this assumption has been experimentally supported [48], it is expected that the product yield will be improved when the production of higher alcohols contributes to faster cell growth. Here, the FBA-based metabolic simulations were performed by maximizing metabolic flux for biomass production from glucose using the linear optimization method. The results revealed that no higher alcohols were produced from glucose in the all modified backbone models of *E. coli*. The low productivity demonstrated by the *in silico *metabolic models mirrors the poor performance of actual recombinant *E. coli *strains just after the introduction of genes required for higher alcohol biosynthesis [4,8,29].

To improve the yield of the target alcohols, the deletion of metabolism-related genes has been demonstrated to regulate the flux balances in the metabolic network [8,11]. The genes, however, must be deleted with regard to both the product yield and cell growth because gene deletion often seriously hampers cell growth. Because the effects of gene deletions could not be predicted empirically, FBA-based metabolic simulations were performed to elucidate the optimal combination of genes to be deleted [49-52].

Via this methodology, the production of higher alcohols by *E. coli *was investigated by performing FBA-based metabolic simulations for all single, double, triple, and quadruple deletion mutants generated from the metabolic model (see Methods for the detailed procedure). In the analysis, reactions instead of genes were removed from the metabolic models. The simulation results were verified by comparisons with literature data. The metabolic engineering of *E. coli *demonstrated that the product yield of a recombinant *E. coli *strain expressing genes for 1-butanol or isobutanol biosynthesis was enhanced by deletions of genes such as ΔadhE, ΔldhA, *ΔfrdBC*, and Δpta as well as ΔadhE, *ΔldhA*, *ΔfrdBC*, and Δpfl, respectively [4,8]. The results were reproduced by FBA-based metabolic simulations using the modified backbone models. The 1-butanol and isobutanol yields were increased to 0.14 and 0.19 (Y_Cmol/Cmol glucose_), respectively, by the deletions of the corresponding reactions.

**Figure 2:**
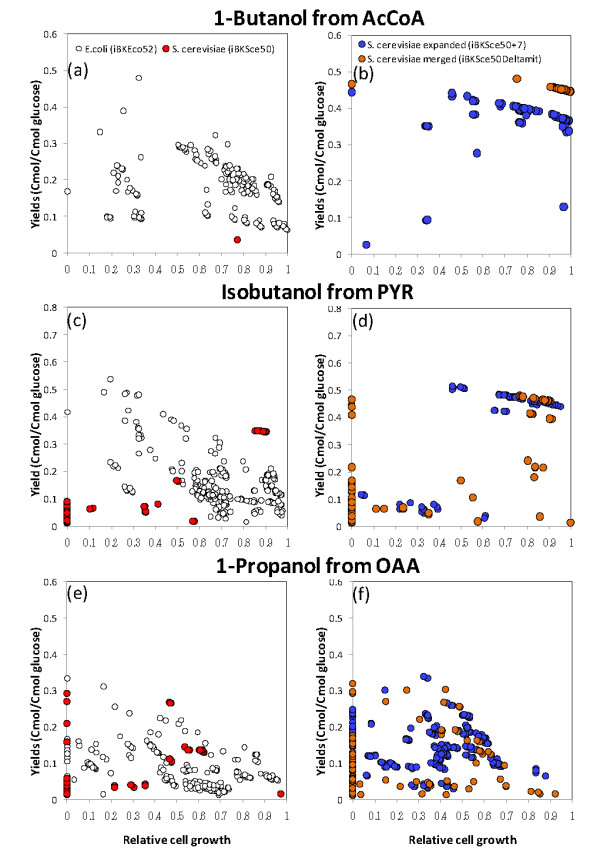
**Metabolic simulations of multiple deletion mutants generated from the backbone models of *E. coli *(iBKEco52) and *S. cerevisiae *(iBKSce50, iBKSce50Δmit, and iBKSce50+7)**. The product yield and cell growth performance of the proper strains are shown for the production of 1-butanol from AcCoA (a and b), isobutanol from PYR (c and d), and 1-propanol from OAA (e and f). Open circles represent the data obtained from *E. coli *(iBKEco50); closed red, blue, and orange circles represent the data of original (iBKSce50), merged (iBKSce50Δmit), and expanded (iBKSce50+7) models of *S. cerevisiae*, respectively. iBKSce50Δmit is an hypothetical *S. cerevisiae *model derived by merging the cytosolic and mitochondrial networks into one compartment. iBKSce50+7 is a model of iBKSce50 expanded by the addition of 7 *E. coli *reactions. The cell growth rate data were represented by relative values. A cell growth rate level determined from the wild type model was arbitrary set at 1.

In the case of the metabolic simulation for 1-butanol production, the growth rates and product yields were predicted for 213,052 multiple deletion models generated from the *E. coli *backbone model. Among the 8,668 viable and 1-butanol-producing models, an elite 347 models were considered "proper strains" in which all deletions contributed to an improvement in the 1-butanol yield (Table 1 and Additional file 4). The performances of the proper strains were compared with respect to the growth speed (x-axis) and 1-butanol yield (y-axis), as shown in Figure 2a. Although a negative correlation between the growth rate and 1-butanol yield was observed, several actively growing strains produce a relatively large amount of 1-butanol. Because these preferable strains are candidate targets for metabolic engineering, these results confirmed that the central metabolism of *E. coli *has promising potential for 1-butanol production as experimentally demonstrated [9,10]. Similar trends were observed for isobutanol (Figure 2c), 1-propanol (Figure 2e), and all other pathways (Additional file 5).

**Table 1:** The numbers of proper strains among all single, double, triple, and quadruple deletion mutants generated from the backbone models for *E. coli* (iBKEco52) and *S. cerevisiae* (iBKSce50, iBKSce50Δmit, and iBKSce50+7)

Target higher alcohols	Number of proper strains Total (single/double/triple/quadruple deletion mutants)			
	
	iBKEco52	iBKSce50	iBKSce50Δmit	iBKSce50+7
1-propanol from OAA	458 (0/11/85/362)	40 (2/16/14/8)	108 (4/27/50/27)	467 (4/23/128/312)
1-Butanol from AcCoA	347 (1/17/87/242)	1 (1/0/0/0)	49 (2/7/17/23)	192 (33/22/44/93)
1-Butanol from OAA	501 (0/10/89/402)	71 (0/10/30/31)	120 (3/25/59/33)	373 (2/17/116/238)
Isobutanol from PYR	501 (1/14/83/403)	38 (2/15/21/0)	85 (4/25/30/26)	276 (2/18/81/175)
3-Methyl-1-butanol from PYR	208 (2/13/51/142)	46 (1/15/21/9)	98 (4/25/46/23)	94 (2/5/33/54)
Isopentenol from AcCoA	330 (0/5/55/270)	0 (0/0/0/0)	104 (0/12/51/41)	166 (2/10/54/100)

The "proper" strains were defined as viable target-producing strains in which all deletions contributed to the improvement of product yields. The growth rates and product yields were determined by FBA-based metabolic simulations. iBKSce50Δmit is an hypothetical *S. cerevisiae *model derived by merging the cytosolic and mitochondrial networks into one compartment. iBKSce50+7 is a model of iBKSce50 expanded by the addition of 7 *E. coli *reactions.

**Figure 3:**
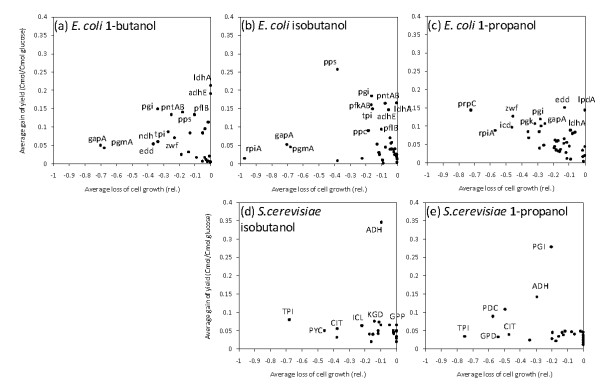
**Effects of the deletion of each reaction in the modified backbone models of *E. coli *(iBKEco52) and *S. cerevisiae *(iBKSce50))**. Average loss of cell growth and average gain of product yields caused by the deletion of each reaction in the modified backbone models of *E. coli *for the production of (a) 1-butanol by AcCoA, (b) isobutanol by PYR, and (c) 1-propanol by OAA are shown in the figure. Effects of the modified backbone models of *S. cerevisiae *for producing (d) isobutanol and (e) 1-propanol are also presented. The results for the production of 1-butanol by AcCoA by *S. cerevisiae *are not shown because there was only 1 target-producing model.

Using the same dataset, the impact of each deletion was evaluated. The improvement in 1-butanol yield and the loss of cell growth caused by each deletion were determined for all proper strains. The effects were averaged for each reaction, and the results are shown in Figures 3a-c. The x- and y-axes represent the average loss of cell growth and average gain of product yield, respectively, caused by deleting the reaction. The results indicated that deletion of genes related to anaerobic fermentation, such as *ldhA *and *adhE*, increased the product yield without decreasing cell growth. Other genes, such as *pgi*, tended to increase the product yield but reduce cell growth. These results rationalize the strategy employed for the metabolic engineering of *E. coli*. After the construction of a metabolic pathway for higher alcohol biosynthesis, the product yields were improved by deleting genes while accounting for the impact on cell growth.

### Metabolic simulation of reaction-deleted mutants of S. cerevisiae

In order to evaluate a performance of the *in silico *analysis using the backbone model of *S. cerevisiae*, iBKSce50, predicted growth rates of all single-reaction-deleted mutants were compared with the experimental data. The published phenotype data demonstrated that an inviable phenotype was observed for single gene-deleted mutants including *Δpgi1*, *Δfba1*, *Δtpi1*, *Δpgk1*, *Δpyk1*, and *Δrki1 *among the genes included in iBKSce50 [53-55]. Most results could be reproduced by the FBA-based metabolic simulation using iBKSce50 (Additional file 6). By using the backbone model of *S. cerevisiae*, iBKSce50, the potential metabolic performance of *S. cerevisiae *was also evaluated using the same procedure for *E. coli*, and the results were compared with that of *E. coli*. The FBA-based metabolic simulations for all single, double, triple, and quadruple deletion mutants indicated that the number of proper strains was smaller than that for *E. coli *for all target alcohols (Table 1). In particular, for the case of acetyl-CoA-derived higher alcohols, no or very few target-producing strains were observed (Figure 2a and Table 1). Exceptionally good results were observed for isobutanol, probably because the redox balance required for isobutanol biosynthesis is similar to that for ethanol (Figure 2c). In the case of the other pathways, such as that towards 1-propanol, actively growing and target-producing strains were not observed among the proper strains (Figure 2e). It was also demonstrated few genes could be deleted without decreasing cell growth (Figure 3d-e). These results suggest that the strategy employed for the engineering of *E. coli *metabolism should not be effective for a construction of *S. cerevisiae *over-producing higher alcohols.

### Properties of the metabolic network

The *in silico *simulations suggest that the central metabolism of *E. coli *could take many states of flux distribution because there are large variations in yield and growth performance among deletion strains (Figures 2a-c). In contrast, the central metabolism of *S. cerevisiae *has rather limited or restricted metabolic behaviors. To support these observations, the natures of *E. coli *and *S. cerevisiae *metabolic networks were compared using elementary mode analysis. The analysis decomposes a complex metabolic network into many pathway subsets comprising a minimal set of enzymes that can support the steady-state operation of cellular metabolism (elementary mode)[33,36,56]. Because 1 elementary mode represents an independent cellular physiological state, the number of generated elementary modes reflects the flexibility of the metabolic network. The numbers of elementary modes of the *E. coli *and *S. cerevisiae *backbone models were 34,880 and 690 respectively, suggesting that the behavior of the central metabolism of *S. cerevisiae *is more restricted than that of *E. coli *(Table 2).

**Table 2:** The network properties of the backbone models for *E. coli* (iBKEco52) and *S. cerevisiae* (iBKSce50, iBKSce50Δmit, and iBKSce50+7)

Network properties	Backbone models			
	
	iBKEco52	iBKSce50	iBKSce50Δmit	iBKSce50+7
Number of nodes	44	50	44	52
Network density	0.117	0.092	0.113	0.095
Characteristic path length	2.577	2.92	2.664	2.790
Number of elementary modes	34,880	690	5,859	25,427

As the difference should be derived from the nature of the metabolic networks, 2 structural properties--network density and characteristic path length--were compared between the *E. coli *and *S. cerevisiae *backbone models [56,57]. Here, a substrate-product pair was considered a pair of nodes connected by an undirected edge. The network density represents the density of reactions among metabolites, and it should be 1 when all metabolites are connected with each other. The observed network densities of the *E. coli *and *S. cerevisiae *backbone models were 0.117 and 0.092, respectively, indicating that the metabolites in the *E. coli *network are more densely connected by the metabolic reactions (Table 2). The characteristic path length indicates the average distances of all of the metabolite-metabolite pairs. For example, the distance between glucose and glucose-6-phosphate is 1 because they are directly connected by the reaction catalyzed by hexokinase. The determined characteristic path lengths suggest that the metabolites in *E. coli *are more closely connected, as the characteristic path length of *E. coli *is smaller than that of *S. cerevisiae *(Table 2). Although a relationship between those properties and higher alcohol production is unclear, the results suggest that the smaller number of elementary modes of *S. cerevisiae *backbone model is, at least in part, derived from the lesser network density and longer characteristic path length of the metabolic network.

### Improvement of the higher alcohol productivity of S. cerevisiae by the addition of *E. coli *genes

To increase the network density and reduce the characteristic path length of the central metabolic network of *S. cerevisiae*, the metabolic structure was modified by considering 2 structural features. Because the metabolic networks of *S. cerevisiae *are compartmentalized into the cytosol and mitochondria, translocations of NADP^+^, NADPH, and acetyl-CoA between these compartments were prohibited in the backbone model (Figure 1). Here, a hypothetical metabolic network of *S. cerevisiae *was constructed by merging the cytosolic and mitochondrial networks and was designated iBKSce50Δmit. The network density and characteristic path length of iBKSce50Δmit were improved to 0.113 and 2.664, respectively, by which the number of elementary modes was also increased to 5,859 (Table 2). Another structural difference between *E. coli *and *S. cerevisiae *is the occurrence of various shortcuts in *E. coli *such as pyridine nucleotide transhydrogenase, the anaplerotic pathways, the Entner-Doudoroff pathway, and acetyl-CoA synthesis by pyruvate formate-lyase. To supplement these metabolic functions in *S. cerevisiae*, the 7 *E. coli *reactions encoded by *pntAB*, *edd*, *pfl*, *pps*, *maeD*, *ppc*, and *mdh *were added to the backbone metabolic model of *S. cerevisiae *as cytosolic reactions. The expanded model was designated as iBKSce50+7. Other reactions, such as those related to the TCA cycle and those catalyzed by large protein complexes, were arbitrarily removed from the inserted reactions. The expanded model exhibited improved network properties and a larger number of elementary modes (Table 2).

**Figure 4:**
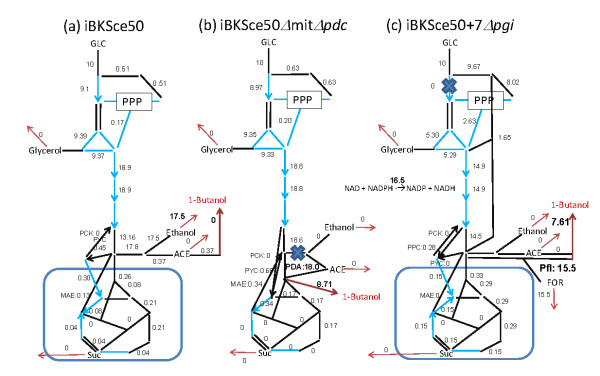
**Optimized flux distribution for 1-butanol synthesis by the gene-deleted *S. cerevisiae *models**. The flux distributions of (a) the original model (iBKSce50), (b) the merged model with the PDC reaction deleted (iBKSce50Δmit*Δpdc*), and (c) the expanded model with the PGI reaction deleted (iBKSce50+7*Δpgi*) are shown in the figure. The oxygen uptake rate is set at 1.0. The steps represented by bold font indicate the reactions playing a role for 1-butanol production.

To test the effects of those modifications, the FBA-based metabolic simulations were performed again using iBKSce50Δmit and iBKSce50+7 (Figure 2b, d and 2f). The results revealed that the metabolic performances of these models were improved in terms of the numbers of proper strains (Table 1) as well as the target production (Figure 2b, d and 2f). For instance, the deletion strains of iBKSce50ΔmitΔ*pdc *(Y_Cmol/Cmol glucose_: 0.445, relative growth rate: 1.0) and iBKSce50+7Δ*pgi *(Y_Cmol/Cmol glucose_: 0.347, relative growth rate: 0.88) were able to produce 1-butanol with better yields than the most of *E. coli *models (Figure 2d). The comparison of the flux distributions at the fixed oxygen consumption rate condition revealed that although the metabolic balance required for 1-butanol biosynthesis could not been attained by the original *S. cerevisiae *model (iBKSce50, Figure 4a), a significant amount of 1-butanol could be generated in the merged model iBKSce50ΔmitΔ*pdc *(Figure 4b) and in the expanded model (iBKSce50+7Δ*pgi*, Figure 4c) by using the modified metabolic functions in these models. Similar preferable results were observed for all pathways investigated (Figure 2d and 2f and Supplementary Data S5).

## Discussion

The comparative *in silico *analyses performed in this study indicated that *E. coli *and *S. cerevisiae *have distinct potentials for higher alcohol production that originate from structural differences in their central metabolic networks (Figure 1). As demonstrated by the FBA-based metabolic analyses, the behavior of the central metabolism of *E. coli *appears more flexible than that of *S. cerevisiae*. Here, the flexibility of the metabolic network indicates the variety of flux distributions taken by the metabolic network. Thus, after the introduction of the genes required for the biosynthesis of higher alcohols, productivity could be improved by gene deletions that restrict the range of flux distributions to produce the target alcohols (Figure 2). However, because the central metabolism in *S. cerevisiae *has limited flexibility, further gene deletions after the introduction of foreign genes usually results in a serious reduction in cell growth (Figure 2 and 3). These results suggest that the strategy employed for the metabolic engineering of *E. coli *would be ineffective for *S. cerevisiae*.

The FBA-based metabolic simulations performed in this study, however, ignored the detailed aspects of metabolic regulations [30,47]. Because the kinetics of enzymatic reactions and the regulation of those activities were not considered, the results of the FBA-based analysis often over- or underestimated the role of metabolic pathways. This means that the FBA-based metabolic simulations could not predict the exact behavior of the metabolism of the microbe. To avoid these problems, metabolic performances derived from central metabolic networks were evaluated by employing the backbone metabolic models. Additionally, the production of higher alcohols in all single, double, triple, and quadruple deletion mutants were comprehensively determined to compare the overall trends of the metabolic performances of *E. coli *and *S. cerevisiae*. A possible drawback of the backbone model is the oversimplification of the metabolic network by omitting important metabolic functions. Although the details of yeast metabolism remain unclear, we reconstructed the backbone model by considering various studies and database informations [35-44].

## Conclusions

If the stylized analysis performed in this study bears any resemblance to the real metabolism of *E. coli and S. cerevisiae*, the implications of this study for metabolic engineering of *S. cerevisiae *are radical. Due to its limited flexibility, the central metabolism of *S. cerevisiae *could not reach the flux distributions required for the effective production of higher alcohols. The comparison of the properties of metabolic network indicated that the cell compartmentalization and the lack of several shortcut reactions are possible causes of the low flexibility (Table 2). This implies that the functionality of the central metabolism of *S. cerevisiae *has to be supplemented by the addition of reactions (Table 2). Thus, the metabolic simulation demonstrated that the modified backbone model of *S. cerevisiae *possessing 7 *E. coli *reactions (iBKSce50+7) and the merged compartment (iBKSce50Δmit) had excellent production capabilities for the target alcohols (Figure 2b, d and 2f). Although the conclusion indicates a theoretical possibility for higher alcohol production, it is suggested that further modification of yeast metabolism requires the introduction of multiple genes and the coordinated regulation of their expression (Figure 4). Although the introduction of a single reaction to the central metabolism of *S. cerevisiae *has been attempted [58,59], an innovative engineering methodology is required for implementing the strategy to the actual *S. cerevisiae *metabolism. This is the next challenge for the synthetic bioengineering of *S. cerevisiae *for the efficient production of higher alcohols.

## Methods

### Model construction

The genome-scale models of *E. coli *(iJR904 GSM/GPR)[45] and *S. cerevisiae *(iMM904)[46] were downloaded from http://gcrg.ucsd.edu. The backbone models of *E. coli *(iBKEco52) and *S. cerevisiae *(iBKSce50) were reconstructed using the information from the literature [35-41] and the databases including EcoCyC (http://ecocyc.org/)[42] and YEASTNET (http://www.comp-sys-bio.org/yeastnet/)[43,44] (Supplementary Data S1). Elementary mode analysis was performed by the aid of METATOOL5.1 [60].

### FBA-based metabolic simulation

The FBA-based metabolic simulation was performed via previously described methods [32] using MATLAB R2010b and glpk version 4.42 to perform the linear programming methods. The metabolic simulations were performed using the following procedure. (1) The linear programming was executed using the reactions for biomass production or growth rate as the objective function. The carbon source was fixed to be glucose consumed at 10 mmol gDW^-1 ^h^-1^ in all analyses. (2) To avoid indefinite results, the flux for target production is minimized under the maximum growth rate condition. (3) The growth rates and target productivities were determined at 16 different oxygen uptake rates (0.1, 1, 2...,15 mmol). Those averages were considered the metabolic performance of the metabolic models. When the levels of growth speed were more than 1% of that of wild type, those deletion mutants were considered viable. The target-producing strains indicated the viable mutants producing target alcohols in yields exceeding Y_Cmol/Cmol glucose _= 0.01. Because most deletions in the target-producing strains are silent or have adverse effects, we defined "proper" strains as target-producing strains in which all deletions contributed to the improvement of product yield. The network density and the characteristic path length were determined with the aid of Network Analyzer version 2.7 [57].

## Competing interests

The authors declare that they have no competing interests.

## Authors' contributions

FM and CF performed the model construction and the metabolic simulations. FM, JI, and TK analyzed the data. SH and AK designed the study. JI, TK, and FM wrote the paper. All authors read and approved the final manuscript.

## Supplementary Material

Additional file 1 **The E. coli backbone model iBKEco52 and the S. cerevisiae backbone model iBKSce50**. Click here for file

Additional file 2 **Fermentation profiles of the *in silico *metabolic models**. Click here for file

Additional file 3 **Pathways for higher alcohol biosyntheses inserted into the backbone models iBKEco52 and iBKSce50**. Click here for file

Additional file 4 **The numbers of viable, target-producing, and proper deletion mutants obtained from all single, double, triple, and quadruple deletion mutants generated from the backbone models of *E. coli *(iBKEco52) and *S. cerevisiae *(iBKSce50, iBKSce50Δmit, and iBKSce50+7)**. Click here for file

Additional file 5 **Metabolic simulations of all single, double, triple, and quadruple deletion mutants generated from the backbone models of *E. coli *(iBKEco52) and *S. cerevisiae *(iBKSce50, iBKSce50Δmit, and iBKSce50+7)**. Click here for file

Additional file 6 **A comparison of the predicted growth rates of all single-reaction-deleted mutants with the experimental data**. Click here for file
