# Supplementary material for: Engineering strategy of yeast metabolism for higher alcohol production
Source: Microb Cell Fact. 2011 Sep 8;10:70. doi: 10.1186/1475-2859-10-70 (PMC3184262; doi:10.1186/1475-2859-10-70)
Supplement: Additional file 1 — The E. coli backbone model iBKEco52 and the S. cerevisiae backbone model iBKSce50. [file 1475-2859-10-70-S1.DOC]

Additional file 1-1 The *E. coli* backbone model iBKEco52.

ptsG; ptsHI; crr GLCext + PEP --> G6P + PYR

pgi G6P = F6P

pfkA; pfkB F6P + ATP --> F16BP + ADP

glpX; fbp F16BP --> F6P

fbaB; fbaA F16BP = DHAP + GA3P

tpiA GA3P = DHAP

gapA GA3P + NAD = 3PGP + NADH

pgk;eno 3PGP + ADP = 3PG + ATP

pgml; ytjC; pgmA 3PG = PEP

pykA; pykF PEP + ADP --> PYR + ATP

pps PYR + ATP --> PEP + AMP

lpdA; aceEF PYR + CoASH + NAD --> ACoA + CO2 + NADH

zwf + pgl G6P + NADP --> 6PG + NADPH

gnd 6PG + NADP --> R5P + CO2 + NADPH

rpe R5P = X5P

rpiA; alsI R5P = RIBO5P

tktAB RIBO5P + X5P = S7P + GA3P

talAB GA3P + S7P = ERY4P + F6P

tktAB ERY4P + X5P = GA3P + F6P

prpC; gltA; acnAB OAA + ACoA --> ICIT + CoASH

icd ICIT + NADP --> AKG + CO2 + NADPH

lpdA; sucAB AKG + NAD + CoASH --> NADH + SCoA + CO2

sucCD SCoA + ADP = SUCC + ATP + CoASH

sdhABCD SUCC + Q --> FUM + QH2

fumABC FUM = MAL

mdh MAL + NAD = OAA + NADH

frdABCD FUM + QH2 --> SUCC + Q

aceA ICIT --> GLYOXY + SUCC

glcB; aceB GLYOXY + ACoA --> MAL + CoASH

ppc PEP + CO2 --> OAA

sfcA; maeB MAL + NAD --> PYR + CO2 + NADH

sfcA; maeB MAL + NADP --> PYR + CO2 + NADPH

pckA OAA + ATP --> PEP + ADP + CO2

pflB; tdcE PYR + CoASH --> ACoA + FOR

poxB PYR + Q --> ACE + CO2 + QH2

ldhA PYR + NADH --> LACext + NAD

adhP; adhE; adhBZM ACoA + 2 NADH --> ETOHext + 2 NAD + CoASH

Pta + ackAB ACoA + ADP --> ACE + CoASH + ATP

edd 6PG --> PYR + GA3P

nuoAHJKLMNEFGBCI; atpABCDEFGHI NADH + 3 ADP + O2ext --> NAD + 3 ATP

cyoABCD; atpABCDEFGHI QH2 + 2 ADP + O2ext --> Q + 2 ATP

ndh NADH + Q = NAD + QH2

pntAB NAD + NADPH = NADP + NADH

OPM1 ATP --> ADP

adk AMP + ATP --> 2 ADP

TRA2 ACE --> ACEext

TRA5 SUCC --> SUCCext

TRA6 FOR --> FORext

TRA7 CO2 --> CO2ext

O2uptake O2e --> O2ext

BIO 0.049 G6P + 0.017 F6P + 0.860 RIBO5P + 1.426 AKG + 2.355 OAA + 0.512 ERY4P + 0.960 PEP + 3.920 PYR + 1.642 3PG + 0.031 GA3P + 1.207 ACoA + 40.680 ATP + 4.079 NAD + 18.320 NADPH + 12.502 NH3ext --> BIOMASS + 1.207 CoASH + 40.680 ADP + 4.079 NADH + 18.320 NADP

Additional file 1-2 The *S. cerevisiae* backbone model iBKSce50.

HXK1-HXK2-GLK1 GLC + ATP --> G6P + ADP

PGI1 G6P <=> F6P

PFK1-PFK2 F6P + ATP --> F16P + ADP

FBP1 F16P --> F6P

FBA1 F16P <=> GA3P + DHAP

TPI1 DHAP <=> GA3P

TDH1-TDH2-TDH3 GA3P + NADcyt <=> P13G + NADHcyt

PGK1 P13G + ADP <=> P3G + ATP

GPM2-GPM3-ENO1 P3G <=> PEP

PYK1-PYK2 PEP + ADP --> PYR + ATP

ZWF1-SOL1 G6P + NADPcyt --> P6G + NADPHcyt

GND1-GND2 P6G + NADPcyt --> RU5P + CO2 + NADPHcyt

RKI1 RU5P <=> R5P

RPE1 RU5P <=> X5P

TKL1 R5P + X5P <=> S7P + GA3P

TAL1 GA3P + S7P <=> F6P + E4P

TKL2 E4P + X5P <=> F6P + GA3P

GPD1-GPD2 DHAP + NADHcyt --> GP + NADcyt

GPP1-GPP2 GP --> GLYC

PDC1-PDC5-PDC6 PYR --> ACA + CO2

ADH1-ADH2-ADH4 ACA + NADHcyt --> ETH + NADcyt

ALD2-ALD6 ACA + NADPcyt --> ACE + NADPHcyt

ACS1-ACS2 ACE + 2 ATP + COA --> ACCOAcyt + 2 ADP

PDA1-PDB1 PYR + NADmit + COA --> NADHmit + CO2 + ACCOAmit

PYC1-PYC2 PYR + CO2 + ATP --> OAA + ADP

CIT1-CIT3-ACO1 ACCOAmit + OAAmit --> ICI + COA

IDP1 ICI + NADmit --> NADHmit + AKG + CO2

IDP3 ICI + NADPmit --> NADPHmit + AKG + CO2

KGD1-KGD2 AKG + NADmit + COA --> SUCCOA + CO2 + NADHmit

LSC1-LSC2 SUCCOA + ADP <=> SUC + ATP + COA

SDH1-SDH2 SUC + FAD --> FUM + FADH2

OSM1 FUM + FADH2 --> SUC + FAD

FUM1 FUM <=> MAL

MDH1 MAL + NADmit <=> OAAmit + NADHmit

MAE1 MAL + NADPmit --> PYR + CO2 + NADPHmit

PCK1 OAA + ATP --> PEP + ADP + CO2

ICL1 ICI --> GLYOX + SUC

MLS1- MLS2 GLYOX + ACCOAmit --> MAL + COA

ShuttleX NADHcyt + NADmit <=> NADcyt + NADHmit

NADHX 24 ADP + 20 NADHmit + 10 O2 --> 24 ATP + 20 NADmit

FADHX 24 ADP + 20 FADH2 + 10 O2 --> 24 ATP + 20 FAD

ATPX ATP --> ADP

OAAmc OAA <=> OAAmit

ACEX ACE --> ACEX

CO2EX CO2 --> CO2EX

PYREX PYR --> PYRX

SUCEX SUC --> SUCX

O2EX O2X --> O2

BIOMASSX 0.002934598 ACCOAmit + 0.024 ACCOAcyt + 0.011 AKG + 0.003 E4P + 0.006 P3G + 0.001 GP + 0.006 PEP + 0.018 PYR + 0.003 R5P + 0.025 G6P + 0.010 OAA + 0.016 NADcyt + 0.006 NADmit + 0.090 NADPHcyt + 0.022 NADPHmit + 1.254 ATP --> 0.1 BIOMASS + 0.016 NADHcyt + 0.006 NADHmit + 0.090 NADPcyt + 0.022 NADPmit + 1.254 ADP + 0.026934598 COA
