# Supplementary material for: Engineering strategy of yeast metabolism for higher alcohol production
Source: Microb Cell Fact. 2011 Sep 8;10:70. doi: 10.1186/1475-2859-10-70 (PMC3184262; doi:10.1186/1475-2859-10-70)
Supplement: Additional file 2 — Fermentation profiles of the in silico metabolic models. [file 1475-2859-10-70-S2.DOC]

Additional file 2. Fermentation profiles of the *in silico* metabolic models. The metabolic simulations were performed using 10 mmol glucose as the carbon source at different oxygen uptake rates/glucose uptake rates. Models: (a) genome-scale model of *E. coli*, iJR904; (b) backbone model of *E. coli*, iBKEco50; (c) genome-scale model of *S. cerevisiae*, iMM904; and (d) backbone model of *S. cerevisiae*, iBKSce52. Because the productions of CO_2_, formate, and acetate were indefinite in the *E. coli* models, those upper and lower limits were shown in the figure.


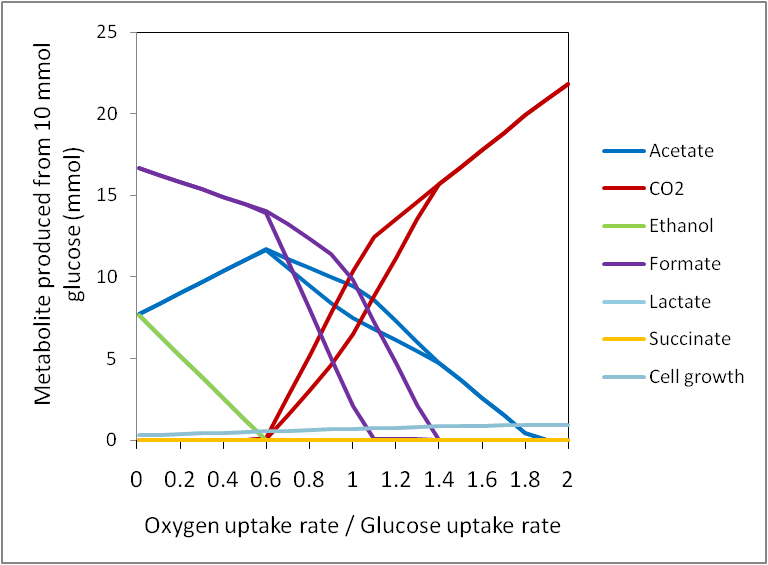

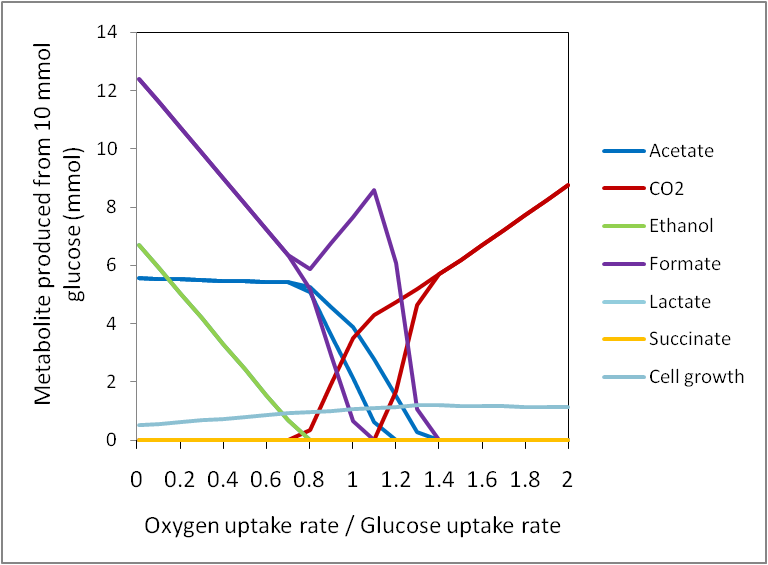


1. iJR904
2. iBKEco50


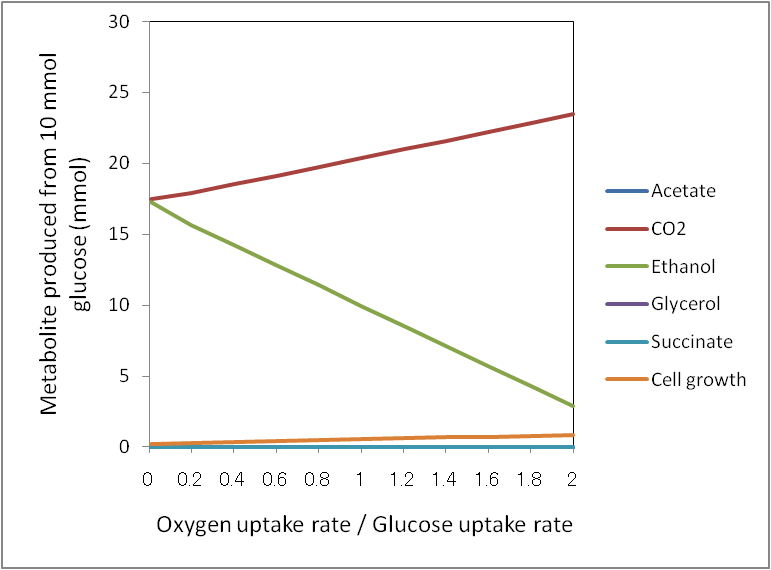

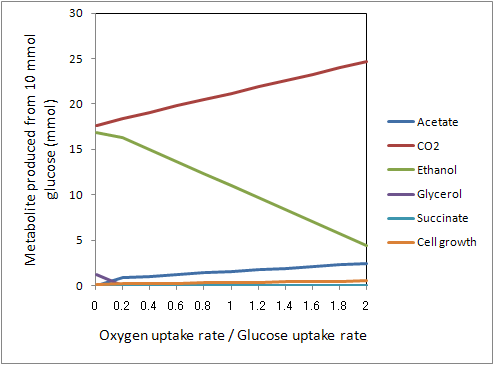


(c) iMM904

1. iBKSce52
