# Supplementary material for: Engineering strategy of yeast metabolism for higher alcohol production
Source: Microb Cell Fact. 2011 Sep 8;10:70. doi: 10.1186/1475-2859-10-70 (PMC3184262; doi:10.1186/1475-2859-10-70)
Supplement: Additional file 3 — Pathways for higher alcohol biosyntheses inserted into the backbone models iBKEco52 and iBKSce50. [file 1475-2859-10-70-S3.DOC]

Additional file 3. Pathways for higher alcohol biosyntheses inserted into the backbone models iBKEco52 and iBKSce50.

| Inserted pathways | Reactions |
| --- | --- |
| 1-Propanol | 1 OAA + 2 ATP + 3 NADPH + 1 NADH  = 1 xNPROext + 2 ADP + 3 NADP + 1 NAD + 1 CO2 |
| 1-Butanol from AcCoA | 2 ACCoA + 4 NADH  = 1 xNBUText + 4 NAD + 2 COA |
| 1-Butanol from OAA | 1 OAA + 2 ATP + 3 NADPH + 1 ACCoA  = 1 xNBUText + 2 ADP + 3 NADP + 3 CO2 + 1 CoASH |
| Isobutanol | 2 PYR + 1 NADPH + 1 NADH  = 1 xIBUText + 1 NADP + 1 NAD + 1 CO2 |
| 3-Methyl-1-butanol | 2 PYR + 1 ACCoA + 1 NADPH  = 1 xMBext + 1 NADP + 3 CO2 + 1 CoASH |
| Isopentenol | 3 ACCoA + 3 ATP + 2 NADPH  = 1 xIPENText + 3 ADP + CO2 + 3 CoASH + 2 NADP |
