# Supplementary material for: Engineering strategy of yeast metabolism for higher alcohol production
Source: Microb Cell Fact. 2011 Sep 8;10:70. doi: 10.1186/1475-2859-10-70 (PMC3184262; doi:10.1186/1475-2859-10-70)
Supplement: Additional file 4 — The numbers of viable, target-producing, and proper deletion mutants obtained from all single, double, triple, and quadruple deletion mutants generated from the backbone models of E. coli (iBKEco52) and S. cerevisiae (iBKSce50, iBKSce50Δmit, and iBKSce50+7). [file 1475-2859-10-70-S4.DOC]

Additional file 4 The numbers of viable, target-producing, and proper deletion mutants obtained from all single, double, triple, and quadruple deletion mutants generated from the backbone models of *E. coli* (iBKEco52) and *S. cerevisiae* (iBKSce50, iBKSce50Δmit, and iBKSce50+7). When the levels of growth speed were more than 1% of that of wild type, those deletion mutants were considered viable. The target-producing strains represented the viable mutants producing target alcohols in yields exceeding Y_Cmol/Cmol glucose_ = 0.01. Because almost all deletions in the target-producing strains had silent or adverse effects, we defined “proper” strains as target-producing strains in which all deletions contributed to the improvement of product yields.

1. The backbone model of *E. coli* (iBKEco52). In total, 213,052 single, double, triple, and quadruple deletion mutants were tested.

|  | Number of mutants | | |
| --- | --- | --- | --- |
| Target compound | Viable | Viable and target-producing | Proper |
| 1-Propanol | 98,030 | 5542 | 458 |
| 1-Butanol from AcCoA | 96,784 | 8677 | 347 |
| 1-Butanol from OAA | 97,951 | 5176 | 501 |
| Isobutanol | 96,779 | 11,825 | 501 |
| 2-Methylbutanol | 96,735 | 11,613 | 208 |
| Isopentenol | 96,777 | 2828 | 330 |

1. The backbone model of *S. cerevisiae* (iBKSce50). In total, 179,446 single, double, triple, and quadruple deletion mutants were tested.

|  | Number of mutants | | |
| --- | --- | --- | --- |
| Target compound | Viable | Viable and target-producing | Proper |
| 1-Propanol | 10,468 | 3204 | 40 |
| 1-Butanol from AcCoA | 9138 | 372 | 1 |
| 1-Butanol from OAA | 9393 | 1086 | 71 |
| Isobutanol | 9629 | 3114 | 38 |
| 2-Methylbutanol | 9400 | 2333 | 46 |
| Isopentenol | 9118 | 0 | 0 |

1. The backbone model of *S. cerevisiae* possessing 7 *E. coli* reactions (iBKSce50Δmit). In total, 317,682 single, double, triple, and quadruple deletion mutants were tested.

|  | Number of mutants | | |
| --- | --- | --- | --- |
| Target compound | Viable | Viable and target-producing | Proper |
| 1-Propanol | 17,525 | 7004 | 108 |
| 1-Butanol from AcCoA | 15,675 | 3815 | 49 |
| 1-Butanol from OAA | 17,208 | 5861 | 120 |
| Isobutanol | 16,160 | 7502 | 85 |
| 2-Methylbutanol | 15,819 | 7123 | 98 |
| Isopentenol | 15,464 | 2293 | 104 |

1. The backbone model of *S. cerevisiae* possessing 7 *E. coli* reactions (iBKSce50+7). In total, 317,682 single, double, triple, and quadruple deletion mutants were tested.

|  | Number of mutants | | |
| --- | --- | --- | --- |
| Target compound | Viable | Viable and target-producing | Proper |
| 1-Propanol | 96,077 | 23,851 | 467 |
| 1-Butanol from AcCoA | 93,728 | 48,152 | 192 |
| 1-Butanol from OAA | 95,676 | 15,169 | 373 |
| Isobutanol | 93,722 | 17,726 | 276 |
| 2-Methylbutanol | 92,035 | 15,721 | 94 |
| Isopentenol | 92,727 | 12,774 | 166 |
