# Supplementary material for: Engineering strategy of yeast metabolism for higher alcohol production
Source: Microb Cell Fact. 2011 Sep 8;10:70. doi: 10.1186/1475-2859-10-70 (PMC3184262; doi:10.1186/1475-2859-10-70)
Supplement: Additional file 5 — Metabolic simulations of all single, double, triple, and quadruple deletion mutants generated from the backbone models of E. coli (iBKEco52) and S. cerevisiae (iBKSce50, iBKSce50Δmit, and iBKSce50+7). [file 1475-2859-10-70-S5.DOC]

Additional file 5 Metabolic simulations of all single, double, triple, and quadruple deletion mutants generated from the backbone models of *E. coli* (iBKEco52) and *S. cerevisiae* (iBKSce50, iBKSce50Δmit, and iBKSce50+7). The product yield and cell growth performance of the reaction-deleted strains were shown for the production of 1-butanol by OAA (a and b), 3-methyl-1-butanol by PYR (c and d), and isopentenol by AcCoA (e and f). Open circles represent the data obtained from *E. coli* (iBKEco50). Closed red, blue and orange circles represent the data of original (iBKSce50), merged (iBKSce50Δmit), and expanded (iBKSce50+7) models of *S. cerevisiae*, respectively.
