# Supplementary material for: Engineering strategy of yeast metabolism for higher alcohol production
Source: Microb Cell Fact. 2011 Sep 8;10:70. doi: 10.1186/1475-2859-10-70 (PMC3184262; doi:10.1186/1475-2859-10-70)
Supplement: Additional file 6 — A comparison of the predicted growth rates of all single-reaction-deleted mutants with the experimental data. [file 1475-2859-10-70-S6.DOC]

Additional file 6. A comparison of the predicted growth rates of all single-reaction-deleted mutants with the experimental data. The growth rate predicted by iBKSce50 (relative to wild type) and the published mutant phenotype data including SGD mutant phenotype data (Engel SR et al: Nucleic acids research 2010, 38:D433-436). Growth rate data from Breslow DK et al. (Nature methods 2008, 5:711-718), and Phenotipic index (PI) of growth rate from PROPHENY database (Warringer J, et al.PNAS 2003, 100:15724-15729.) are shown in the table. nd, not determined.

| Removed reaction | Growth rate predicted by iBKSce50 (Relative to wild type) | Delete gene | SGD Mutant Phenotype Data | Phenotipic index (PI) of Growth Rate from PROPHENY database | Growth Rate from Breslow et al.(Relative to wild type) |
| --- | --- | --- | --- | --- | --- |
| HXK1-HXK2-GLK1 | 0 | HXK1 | viable | -0.015 | 0.998 |
|  |  | HXK2 | viable | -0.135 | 0.861 |
|  |  | GLK1 | viable | -0.057 | 1.005 |
| PGI1 | 0 | PGI1 | inviable | nd | nd |
| PFK1-PFK2 | 0 | PFK1 | viable | -0.218 | nd |
|  |  | PFK2 | viable | -0.056 | 0.833 |
| FBP1 | 1.00 | FBP1 | viable | 0.025 | 1.003 |
| FBA1 | 0 | FBA1 | inviable | nd | nd |
| TPI1 | 0.56 | TPI1 | inviable | 0.059 | nd |
| TDH1-TDH2-TDH3 | 0 | TDH1 | viable | -0.094 | nd |
|  |  | TDH2 | viable | 0.041 | 1.012 |
|  |  | TDH3 | viable | -0.028 | 0.943 |
| PGK1 | 0 | PGK1 | inviable | nd | nd |
| GPM-ENO | 0 | GPM2 | viable | -0.084 |  |
|  |  | GPM3 | viable | 0.053 | 1.002 |
|  |  | ENO1 | nd | 0.031 | 1.014 |
| PYK1-PYK2 | 0 | PYK1 | inviable | nd | nd |
|  |  | PYK2 | viable | 0.008 | 1.003 |
| ZWF1-SOL1 | 1.00 | ZWF1 | viable | -0.380 | 0.604 |
|  |  | SOL1 | viable | 0.004 | 0.993 |
| GND1-GND2 | 1.00 | GND1 | viable | -0.048 | nd |
|  |  | GND2 | viable | -0.008 | 1.003 |
| RKI1 | 0 | RKI1 | inviable | nd | nd |
| RPE1 | 1.00 | RPE1 | viable | -0.043 | 0.916 |
| TKL1 | 1.00 | TKL1 | viable | -0.114 | 0.901 |
| TAL1 | 1.00 | TAL1 | viable | -0.014 | 1.024 |
| GPD1-GPD2 | 0 | GPD1 | viable | 0.014 | nd |
|  |  | GPD2 | viable | -0.172 | 0.994 |
| GPP1-GPP2 | 1.00 | GPP1 | viable | -0.507 | 0.941 |
|  |  | GPP2 | viable | nd | 1.005 |
| PDC1-PDC5-PDC6 | 0 | PDC1 | viable | 0.014 | 0.950 |
|  |  | PDC5 | viable | 0.009 | 0.986 |
|  |  | PDC6 | viable | 0.012 | 0.942 |
| ADH1-ADH2-ADH4 | 0.82 | ADH1 | viable | -0.624 | 0.938 |
|  |  | ADH2 | viable | 0.091 | 1.000 |
|  |  | ADH4 | viable | 0.009 | 0.881 |
| ALD2-ALD6 | 0 | ALD2 | viable | 0.009 | 1.000 |
|  |  | ALD6 | viable | -0.136 | 0.807 |
| ACS1-ACS2 | 0 | ACS1 | viable | 0.030 | 0.990 |
|  |  | ACS2 | inviable | nd | nd |
| PDA1-PDB1 | 0 | PDA1 | viable | -0.114 | 0.945 |
|  |  | PDA2 | viable | -0.120 | 0.956 |
| PYC1-PYC2 | 1.00 | PYC1 | viable | 0.002 | 1.000 |
|  |  | PYC2 | viable | -0.152 | 0.983 |
| CIT1-CIT3-ACO1 | 0 | CIT1 | viable | 0.023 | 0.989 |
|  |  | CIT3 | viable | 0.010 | 1.002 |
|  |  | ACO1 | viable | -0.012 | nd |
| IDP1 | 0.98 | IDP1 | viable | -0.016 | 1.002 |
| IDP3 | 0.99 | IDP3 | viable | -0.010 | 1 |
| KGD1-KGD2 | 0.97 | KGD1 | viable | -0.026 | 0.822 |
|  |  | KGD2 | viable | -0.144 | 0.990 |
| LSC1-LSC2 | 0.97 | LSC1 | viable | 0.007 | 0.975 |
|  |  | LSC2 | viable | 0.004 | 0.997 |
| SDH1-SDH2 | 0.96 | SDH1 | viable | 0.075 | 1.000 |
|  |  | SDH2 | viable | -0.037 | 1.004 |
| OSM1 | 1.00 | OSM1 | viable | 0.031 | 1.015 |
| FUM1 | 0.96 | FUM1 | viable | 0.000 | 0.978 |
| MDH1 | 0.96 | MDH1 | viable | 0.043 | 1.005 |
| MAE1 | 1.00 | MAE1 | viable | -0.017 | 1.003 |
| PCK1 | 1.00 | PCK1 | viable | 0.000 | 0.968 |
| ICL1 | 1.00 | ICL1 | viable | -0.004 | 1.004 |
| MLS1-2 | 1.00 | MLS1 | viable | -0.022 | 0.986 |
|  |  | MLS2 | viable | nd | nd |
